# A Comparative Analysis of SARS-CoV-2 Variants of Concern (VOC) Spike Proteins Interacting with hACE2 Enzyme

**DOI:** 10.3390/ijms25158032

**Published:** 2024-07-23

**Authors:** Jiawei Chen, Lingtao Chen, Heng Quan, Soon Goo Lee, Kaniz Fatama Khan, Ying Xie, Qiaomu Li, Maria Valero, Zhiyu Dai, Yixin Xie

**Affiliations:** 1 College of Computing, Data Science and Society, University of California, Berkeley, CA 94720, USA; jc01@berkeley.edu; 2 College of Computing and Software Engineering, Kennesaw State University, Marietta, GA 30060, USA; lchen25@students.kennesaw.edu (L.C.); yxie2@kennesaw.edu (Y.X.); qli12@students.kennesaw.edu (Q.L.); mvalero2@kennesaw.edu (M.V.); 3 Department of Civil and Urban Engineering, New York University, Brooklyn, NY 10012, USA; hq322@nyu.edu; 4 Department of Molecular and Cellular Biology, Kennesaw State University, Kennesaw, GA 30144, USA; slee295@kennesaw.edu; 5 Department of Chemistry and Biochemistry, Kennesaw State University, Kennesaw, GA 30144, USA; kkhan12@students.kennesaw.edu; 6 Division of Pulmonary and Critical Care Medicine, John T. Milliken Department of Medicine, Washington University School of Medicine in St. Louis, St. Louis, MO 63110, USA; zhiyudai@arizona.edu

**Keywords:** COVID-19, coronavirus, spike protein, angiotensin-converting enzyme 2, hACE2, protein–protein interactions, binding affinity, molecular dynamics, salt bridge, hydrogen bonds

## Abstract

In late 2019, the emergence of a novel coronavirus led to its identification as SARS-CoV-2, precipitating the onset of the COVID-19 pandemic. Many experimental and computational studies were performed on SARS-CoV-2 to understand its behavior and patterns. In this research, Molecular Dynamic (MD) simulation is utilized to compare the behaviors of SARS-CoV-2 and its Variants of Concern (VOC)-Alpha, Beta, Gamma, Delta, and Omicron-with the hACE2 protein. Protein structures from the Protein Data Bank (PDB) were aligned and trimmed for consistency using Chimera, focusing on the receptor-binding domain (RBD) responsible for ACE2 interaction. MD simulations were performed using Visual Molecular Dynamics (VMD) and Nanoscale Molecular Dynamics (NAMD2), and salt bridges and hydrogen bond data were extracted from the results of these simulations. The data extracted from the last 5 ns of the 10 ns simulations were visualized, providing insights into the comparative stability of each variant’s interaction with ACE2. Moreover, electrostatics and hydrophobic protein surfaces were calculated, visualized, and analyzed. Our comprehensive computational results are helpful for drug discovery and future vaccine designs as they provide information regarding the vital amino acids in protein-protein interactions (PPIs). Our analysis reveals that the Original and Omicron variants are the two most structurally similar proteins. The Gamma variant forms the strongest interaction with hACE2 through hydrogen bonds, while Alpha and Delta form the most stable salt bridges; the Omicron is dominated by positive potential in the binding site, which makes it easy to attract the hACE2 receptor; meanwhile, the Original, Beta, Delta, and Omicron variants show varying levels of interaction stability through both hydrogen bonds and salt bridges, indicating that targeted therapeutic agents can disrupt these critical interactions to prevent SARS-CoV-2 infection.

## 1. Introduction

At the end of 2019, a novel coronavirus designated as severe acute respiratory syndrome coronavirus 2 (SARS-CoV-2), which is highly transmissible, emerged in the city of Wuhan, China, and caused an outbreak called coronavirus disease (COVID-19) [1,2]. Unlike the closely related SARS-CoV in the 2003 outbreak, symptom-based screening alone could not contain it [3]. It is the third human coronavirus known to co-opt the peptidase angiotensin-converting enzyme 2 (ACE2) to enter the cell [4]. This SARS-CoV-2 is a positive-sense, single-stranded RNA virus that is rapidly evolving and continually accrues genomic mutations as it continues to be transmitted [5]. Genetic mutations of SARS-CoV-2 will occur by two mechanisms: randomly occurring mutations followed by selection and recombination [6]. More than one mutation type can be found at the same position in the spike protein sequence [7]. Global Initiative Sharing Avian Influenza Data (GISAID) provides the names for the SARS-CoV-2 lineage; this scientific nomenclature is complex for ordinary people to remember or recall. As a result, people started calling these variants by the place where they were detected. To simplify this, the WHO Virus Evolution Working Group has recommended using letters of the Greek alphabet for naming variants of SARS-CoV-2 [8]. These highly mutated forms of SARS-CoV-2 had enhanced transmission rates relative to previous variants and were termed *Variants of Concern* (VOCs)—designated Alpha, Beta, Gamma, Delta, and Omicron [2,8]. 

The Alpha variant of the COVID-19 virus (B.1.1.7) was first identified in the United Kingdom in September 2020 [9]. Within one month, two rapidly growing lineages with large numbers of genetic changes were reported from South Africa [10] and Brazil [11]. The B.1.351 (Beta) variant rose in prevalence in South Africa from 11% in October to 87% by December [12]. The P.1 (Gamma) variant emerged in Manaus, Brazil, a region estimated to have achieved an infection rate approaching 75% by October 2020 but experienced a surge in new cases beginning in November 2020 [13]. Subsequently, the Delta variant (B.1.617.2) increased in prevalence from 2% in February 2021 to 87% in May 2021 in Maharashtra, India, as India experienced a dramatic surge in cases [14]. Later on, The Omicron variant of SARS-CoV-2 (B.1.1.529) was first identified in South Africa and Botswana and was reported to the World Health Organization (WHO) on November 24, 2021 [15]. The emergence of these variants highlights the ongoing evolution of SARS-CoV-2. Continued surveillance and monitoring of the virus’s genetic changes will be crucial in controlling the ongoing pandemic and developing effective treatments and vaccines [16].

Experimental studies have revealed that spike proteins of both SARS-CoV-2 and SARS-CoV bind to ACE2 before entering the cell for replication [17]. Traditional wet-lab protein structural studies techniques involved nuclear magnetic resonance, X-ray crystallography, and cryo-electron microscopy [18]. Those traditional methods were widely used before because they can investigate the dynamics and flexibility of molecules in their natural solution state and detailed views of molecular interactions. But most of the techniques are restricted by size limitations, lab setting requirements, budgets, time consumption, etc. The structural analysis finds out the different parts of mutation among those variants. The structural comparison illustrates that some variants have mutations that occurred in some locations different than the others, and some of the variants have fewer mutations while some variants have more mutations.

Many computational methods are used to study SARS-CoV-2 and its variants [19,20,21,22,23,24,25,26,27,28,29,30,31,32,33,34,35,36,37,38,39,40,41,42,43,44]. For example, Khan revealed that the B.1.618 variant is an antibody-escaping variant with slightly altered ACE2-RBD affinity from the perspectives of binding affinity and hydrogen bonding network [45]. Kumar compared the spike proteins of Omicron and Delta variants. He observed a disorder–order transition in the Omicron variant between spike protein RBD regions 468–473, which may significantly influence disordered residues/regions on spike protein stability and binding to ACE2 [46]. These analyses will help researchers to understand the structural and binding changes in the different domains and to assess the future repercussions of these changes. AlphaFold is a computational tool in the protein structural domain that can be used in protein structure prediction [47,48]. The original virus and variants like Delta and Omicron sequences were predicted using I-Mutant3.0, a support vector machine-based tool for predicting protein stability changes resulting from single point mutations [46]. Protein modeling, structure-based virtual screening, and comparative docking are the other methods used [49]. Moreover, electrostatic intermolecular interactions are important and have been studied by different research groups [50,51], including pH-dependency potential and energy of association of spike protein RBDs and hACE2 [52,53].

In this research, computational techniques, including Molecular Dynamic Simulation (MD simulation) and protein-protein interaction and analysis, are utilized to study the interaction between the spike protein of SARS-CoV-2 and its variants (Alpha, Beta, Gamma, Delta, and Omicron) and the human ACE2 protein. Tools like NAMD2, VMD, Chimera, and Prodigy aided in aligning structures, comparing mutations, performing MD simulations, and extracting salt bridges and hydrogen bonds’ data from the results of MD simulations. Our analysis concludes that the Original and Omicron are most structurally alike. The Gamma variant forms the strongest interaction with hACE2 through hydrogen bonds, and Omicron easily attracts the hACE2 receptor. The Original, Beta, Delta, and Omicron variants show varying levels of interaction stability through hydrogen bonds and salt bridges, indicating that targeted therapeutic agents can disrupt these critical interactions to prevent SARS-CoV-2 infection.

## 2. Results and Discussion

### 2.1. Structure Comparison

**Figure 1:**
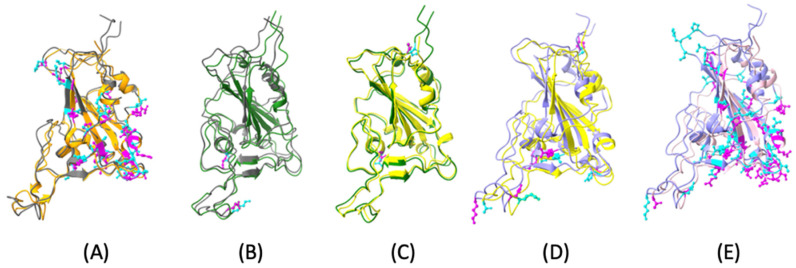
Structure comparisons between spike (S) protein Receptor-Binding Domains (RBDs) of Original SARS-CoV-2 and its variants. (**A**) Original (orange) and Alpha (gray) (Root Mean Square Deviation (RMSD) 1.44 Å); (**B**) Alpha (gray) and Beta (green) (RMSD 1.48 Å); (**C**) Beta (green) and Gamma (yellow) (RMSD 1.09 Å); (**D**) Gamma (yellow) and Delta (purple) (RMSD 0.96 Å); (**E**) Delta (Purple) and Omicron (baby pink) (RMSD 0.73 Å) with their mutations (cyan and magenta).

The mutations were identified by comparing the sequences of each protein, and we colored the sequence IDs where differences were found. We compared these variants chronologically (e.g. Original vs. Alpha vs. Beta). The second highest number of mutations is between the Original and Alpha variants (Figure 1A). Only two mutations exist between the Alpha and Beta variants (Figure 1B) or the Beta and Gamma variants (Figure 1C). The third highest is between Gamma and Delta (Figure 1D). Notably, most mutations occurred between the Delta variant and the Omicron variant (Figure 1E).

With the same color of each variant shown above, we noticed that in most variants, the original protein structure has many mutations among these variants. The Alpha and Beta, Beta and Gamma structural comparisons show fewer mutations than other variants. Next, we visualized the protein structures among the original SARS-CoV-2 and other variants.

The RMSD values indicate how similar the two structures are. Using an online tool 2StrucCompare (http://2struccompare.cryst.bbk.ac.uk/index.php, accessed on 1 February 2023), we calculated the RMSD value between Original SARS-CoV-2 and the Alpha variant (1.44 Å), the Alpha and Beta variants (1.48 Å), the Beta and Gamma variants (1.09 Å), the Gamma and Delta variants (0.96 Å), and the Delta and Omicron variants (0.73 Å). The RMSD values of Original SARS-CoV-2 protein and its variants are as follows: Original and Alpha is 1.44 Å; Original and Beta is 0.99 Å; Original and Gamma is 0.70 Å; Original and Delta is 0.79 Å; and Original and Omicron is 0.45 Å. Therefore, the Original and Omicron variants are the two most structurally similar proteins.

### 2.2. Hydrogen Bonds

**Figure 2:**
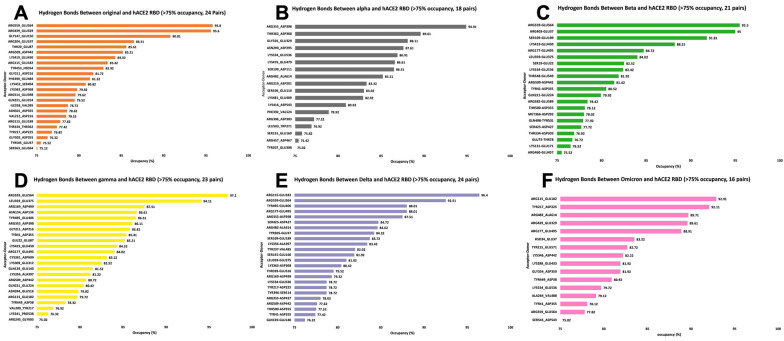
Hydrogen bonds analysis between spike proteins and hACE2 RBD with occupancy over 75%. (**A**) Original (orange); (**B**) Alpha (gray); (**C**) Beta (green); (**D**) Gamma (yellow); (**E**) Delta (purple); (**F**) Omicron (pink). Hydrogen bonds with occupancies > 75% were considered for the analysis. The title of each graph shows the number of hydrogen bonds formed during the interaction of the variant with hACE2 and having occupancy > 75%. Each bar in these charts describes the hydrogen bond Acceptor–Donor pair with its occupancy in %.

Based on the MD simulation, the hydrogen bonds at S protein RBDs and hACE2 were calculated, as shown in Figure 2. Figure 2 depicts the hydrogen bonds between the interaction of Original, Alpha, Beta, Gamma, Delta, and Omicron variants with hACE2. Each interaction of variants has 24, 18, 21, 23, 24, and 16 pairs of hydrogen bonds with occupancies more than 75%, with the highest occupancies being 95.8%, 94.91%, 95.5%, 97.2%, 96.4%, and 92.91% for each variant. This shows that Gamma has the highest occupancy among all the variants. The hydrogen bond pairs of Arginine amino acid (Arg) and Glutamic amino acid (Glu) with the highest occupancies are Arg559-Glu564, Arg115-Glu182, and Arg559-Glu564. If the cutoff was picked at 90%, these variants have 3, 1, 3, 2, 2, and 2 hydrogen bonds; this shows that Original and Beta form the highest number of hydrogen bond pairs with occupancy more significant than 90%, followed by the Gamma, Delta, and Omicron variants. This observation strongly suggests that hydrogen bonding significantly contributes to the overall stability of these protein-protein interactions.

Notably, among the hydrogen bond acceptors, the Arginine amino acid consistently appears in all variants, forming hydrogen bonds with high occupancy. Also, the Glutamic amino acid, especially Glu564, is the donor in most high-occupancy hydrogen bonds. Arginine and Aspartic acid are the pivotal amino acids identified in forming these high-occupancy hydrogen bonds across all variants. Their presence at the interaction interfaces signifies their critical role in defining the binding affinity of the viral spike protein to the human cell receptor hACE2.

### 2.3. Salt Bridges

**Figure 3:**
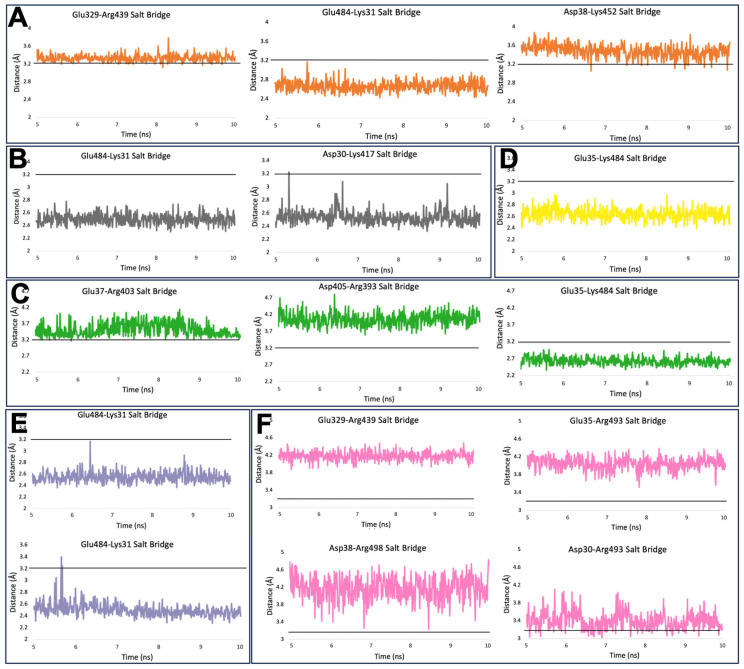
Formation of salt bridges between hACE2 and spike proteins. (**A**) Original (orange); (**B**) Alpha (gray); (**C**) Beta (green); (**D**) Gamma (yellow); (**E**) Delta (purple); (**F**) Omicron (pink). Each panel depicts the distance in units of Å between donor (left) and acceptor (right) amino acids over time in units of ns. The cutoff is set to 3.2 Å and shown in the black reference line of each graph.

Figure 3 depicts the salt bridges formed between the S protein of SARS-CoV-2 and its variants with hACE2. From the data obtained from the MD simulation, only the last 500 frames were considered for the analysis, as the interaction is unstable during the first 500 frames. The standard line at 3.2 Å for all the salt bridges in Figure 3 is used to determine the strength of the salt bridge. If the distance is consistently above the classic line, it is considered a weak salt bridge, whereas if the space is consistently below the standard line, it is regarded as a strong salt bridge. From Table 1, Original, Alpha, Beta, Gamma, Delta, and Omicron have 1, 2, 1, 1, 2, and 1 pair(s) of stronger salt bridges and 2, 0, 2, 0, 0, and 3 pair(s) of weaker salt bridges, respectively; this shows that the Alpha and Delta variants have the highest number of solid salt bridges and Gamma exhibits weaker interaction among all the variants because it has only one pair of strong salt bridge and there are no weak salt bridges.

Further deepening the complexity, our study also examined the specific amino acid pairs responsible for forming these salt bridges. Glutamic acid (Glu) and Lysine (Lys) emerged as the predominant pair, with Glutamic acid frequently functioning as the donor in these interactions and Lysine acting as the acceptor in all the stable salt bridges. Intriguingly, fewer sturdy bridges were formed when Glutamic acid was paired with Arginine, despite Arginine’s role as an acceptor. This observation adds another layer to our understanding of the structural biology of these variants, indicating that not all amino acids contribute equally to the stability of salt bridges and, by extension, to the overall structural integrity of the protein.

**Figure 4:**
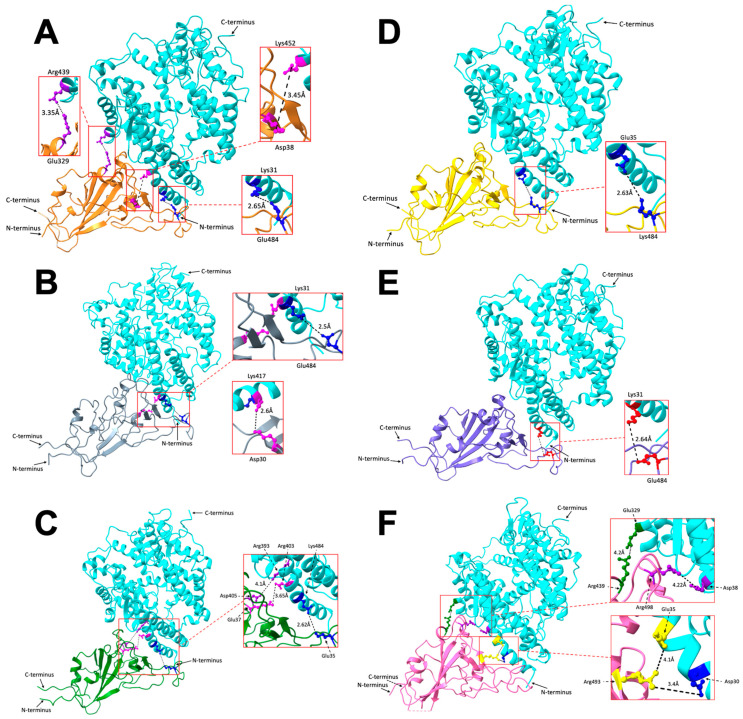
Salt bridge amino acid visualization between (**A**) Original (orange) and hACE2 (cyan), three pairs (Glu329-Arg439, Asp38-Lys452, Glu484-Lys31); (**B**) Alpha (grey) and hACE2, two pairs (Asp30-Lys417, Glu484-Lys31); (**C**) Beta (green) and hACE2, three pairs (Asp405-Arg393, Glu37-Arg403, Glu35-Lys484); (**D**) Gamma (yellow) and hACE2, one pair (Lys484-Glu35); (**E**) Delta (purple) and hACE2, one pair (Glu484-Lys31); (**F**) Omicron (pink) and hACE2, four pairs (Arg439-Glu329, Arg498-Asp38, Arg493-Glu35, Arg493-Asp30).

All the salt bridges we analyzed in Figure 3 are visualized in the protein structure, as shown in Figure 4. The hACE2 receptors are colored in cyan, spike proteins: Original colored in orange, Alpha colored in gray, Beta colored in green, Gamma colored in yellow, Delta colored in purple, and Omicron colored in pink. Amino acid pairs are colored in different colors for different pairs between hACE2 receptor and spike proteins.

The Original, Alpha, Beta, Gamma, Delta, and Omicron variants have 1, 2, 1, 1, 2, and 1 pair(s) of stronger salt bridges and 2, 0, 2, 0, 0, and 3 pairs of weaker salt bridges, respectively; this shows that Alpha and Delta form the more robust interaction with hACE2. When variants with less strong salt bridges are compared, like Original, Beta, Gamma, and Omicron, Gamma exhibits weaker interaction because it has no weak salt bridges, while the other variants have few.

### 2.4. Electrostatic Calculations

**Figure 5:**
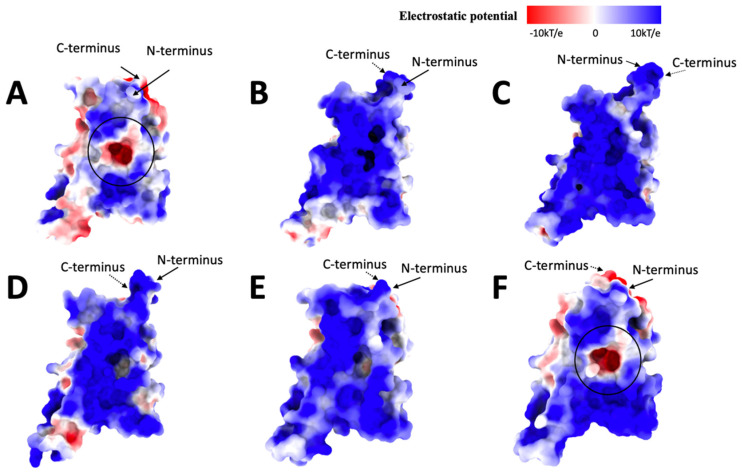
Electrostatic surface (front) of (**A**): Original, (**B**): Alpha, (**C**): Beta, (**D**): Delta, (**E**): Gamma, (**F**): Omicron. The electrostatic potentials are red for negative potential, white for neutral potential, and blue for positive potential, ranging from −10 kT/e to 10 kT/e. The black circles indicate the difference in shape among all variants.

Original (Figure 5A) has a more even electrostatic potential distribution, while the positive potential is dominant in distribution; there are some neutral potential and negative potential in the middle of the front view surface and some on the sides, mainly on top and bottom. Alpha (Figure 5B) has more positive potential in the front view of the electrostatic surface and some negative and neutral potential on the side compared to the Original. The neutral potential is the second dominant potential in Alpha. Beta (Figure 5C) is mainly covered by positive potential, while some minor neutral and negative potential are on the side. Delta (Figure 5D) is dominated by positive potential. The negative potential is the second dominant, combined with minor neutral potential. Gamma (Figure 5E) is dominated by positive potential, with some neutral potential on the surface and some negative potential on the side. Omicron (Figure 5F) is dominated by positive potential, with more negative potential in the middle of the front view and top combined with some neutral potential (marked with a circle in Figure 5F). The other variants have a similar electrostatic potential distribution for the front view except for the Original and Omicron variants. The Original and Omicron both have negative and minor neutral potential on the side of the middle part of the front view, which shows high similarities between them. Note that the bottom area is the binding side.

**Figure 6:**
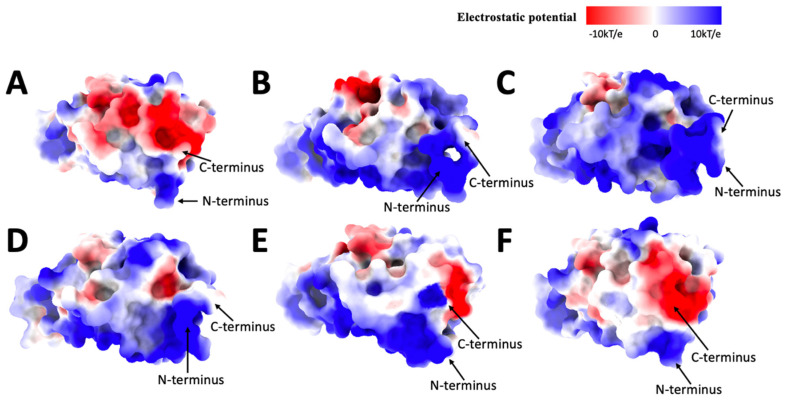
Electrostatic surface (top) of (**A**): Original, (**B**): Alpha, (**C**): Beta, (**D**): Delta, (**E**): Gamma, (**F**): Omicron. The electrostatic potentials are red for negative potential, white for neutral potential, and blue for positive potential.

In the top view of the Original (Figure 6A), we can see that the negative potential is more dominant than the positive and neutral potential. The top view of Alpha (Figure 6B) is dominated by neutral and positive potential, and some red possibilities are on the side and center. The top view of Beta (Figure 6C) is mainly dominated by positive and neutral potentials, with some minor weak negative potential on the side. Delta (Figure 6D) combines positive, negative, and neutral possibilities while the negative potentials are scattered. The top view of the center of the Gamma variant (Figure 6E) has more neutral potential, and the positive and negative potential are on the side. Omicron distributes the positive, neutral, and negative potentials more evenly.

The top view of the electrostatic surfaces shows that Original and Omicron have similar electrostatic potential distributions, which are more likely dominated by negative potential. The Alpha, Beta, and Delta variants have similar electrostatic potential distributions because they are more likely to be dominated by the positive potential. The Gamma variant has a more even electrostatic potential distribution.

**Figure 7:**
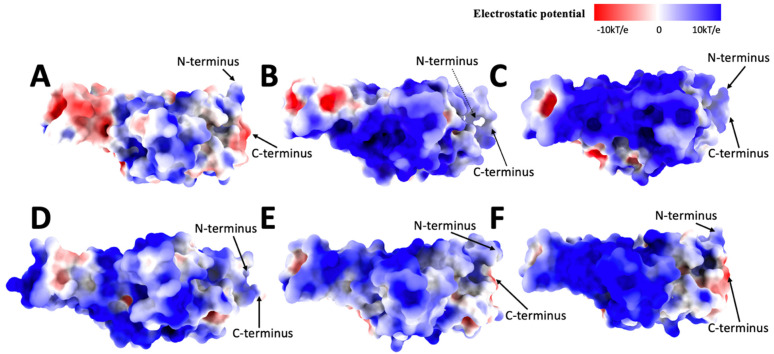
Electrostatic surface (bottom) of (**A**): Original, (**B**): Alpha, (**C**): Beta, (**D**): Delta, (**E**): Gamma, (**F**): Omicron. The electrostatic potentials are red for negative potential, White for neutral potential, and Blue for positive potential.

Moving over to the bottom view of the electrostatic surface of these variants, which are the binding sites of these variants. The original (Figure 7A) has a more even electrostatic potential distribution; half of the bottom (the receptor part) is dominated by negative potential along with some neutral potential, and the other half of the base is dominated by positive potential. The bottom part of the Alpha variant electrostatic potential (Figure 7B) is dominated by positive potential. In contrast, the receptor part is covered by negative and neutral potential on the side. Beta (Figure 7C) is mainly covered by positive potential, but there are some negative potentials in the receptor part and the bottom of graph C. The bottom view of the Delta electrostatic potential (Figure 7D) differs from the previous variants; Delta is covered mainly by positive electrostatic potential, and even the receptor part is entirely covered by positive potential. Gamma (Figure 7E) is also mostly covered by the positive potential. Still, there are some weak negative potentials in the receptor part, some neutral potentials, and the bottom of Graph E. Omicron (Figure 7F) is dominated by positive potential, but the receptor part is covered by neutral and weak negative potential.

According to our previous research results [33,35,36,40], human ACE2 is overall negative, so if the electrostatic potential of these variants is generally dominated by positive potential, that means the binding affinity between the human ACE2 and the variant is high, which means the humans are more easily to be affected by the variant.

Interestingly, the pocket (leftmost outgoing part on each graph) is an active mutation site. By comparing the Original and the Delta variants, the pocket area has more negative potential in Original than in Delta. Then, from the Delta to Gamma variants, the electrostatic potential becomes negative potential again, and from the Gamma to Omicron variants, the pocket is neutral with slightly negative potential. Because the pocket is targeted in drug design, these observations may relate to drug effectiveness design.

### 2.5. Hydrophobic Analysis

**Figure 8:**
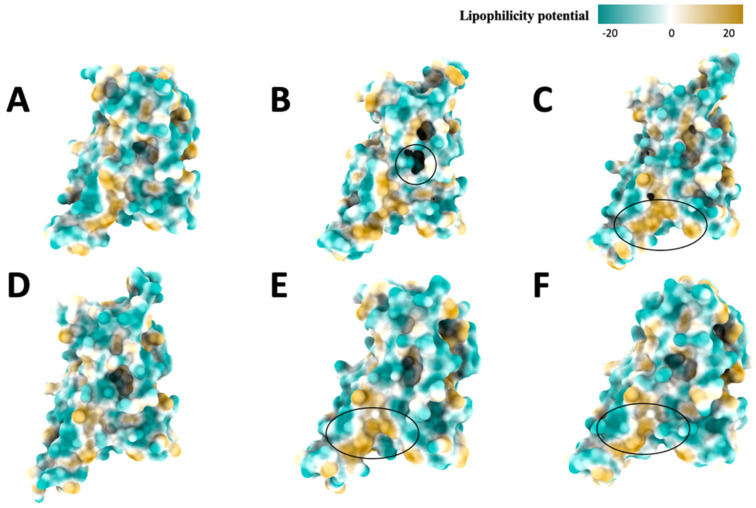
Hydrophobic (front) view of spike proteins, (**A**): Original, (**B**): Alpha, (**C**): Beta, (**D**): Delta, (**E**): Gamma, (**F**): Omicron. The color changes from yellow to green to represent the amino acids lipophilicity potential, ranging from 20 to −20. The circles show the structural differences and lipophilicity potential distribution differences.

Since the hydrophobic surface mainly measures the hydrophobicity of amino acid side chains. We can see that the original variant’s (Figure 8A) hydrophobic distribution has more negative lipophilicity potential, but in general, the distribution looks random and does not have a specific pattern. We notice that the distribution of positive and negative lipophilicity potentials and some neutral lipophilicity potentials are like the Alpha (Figure 8B) hydrophobic surface. Notice that the shadow of the protein structure causes the black hole region in the center of graph B. The Beta variant’s (Figure 8C) negative lipophilicity potential is dominant, but some positive lipophilicity potentials are near the bottom. For the Delta variant (Figure 8D), the overall distribution is still dominated by negative lipophilicity potential. For the Gamma variant (Figure 8E), the overall hydrophobic distribution is dominated by the negative potential, with some positive potentials near the bottom. For Omicron (Figure 8F), the lipophilicity distribution is dominated by negative lipophilicity potential.

By eyeballing, the lipophilicity distribution of the Beta (Figure 8C), Gamma (Figure 8D), and Omicron (Figure 8F) variants are similar, which means they also have similar hydrophilic properties. These hydrophobic surfaces are consistent with the previous electrostatic surfaces; the strong electrostatic potential tends to be more hydrophilic due to interactions with water.

**Figure 9:**
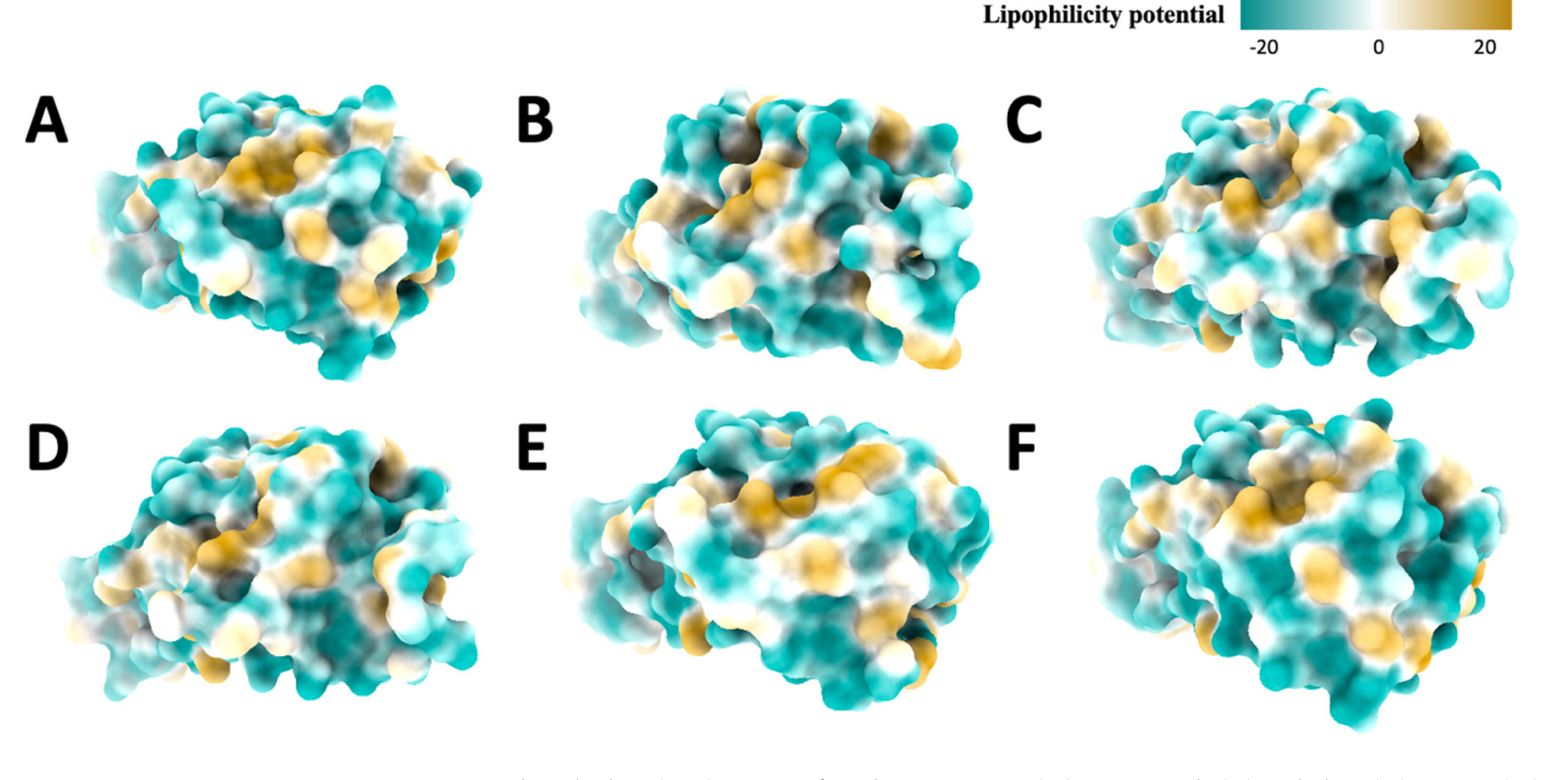
Hydrophobic (top) view of spike proteins, (**A**): Original, (**B**): Alpha, (**C**): Beta, (**D**): Delta, (**E**): Gamma, (**F**): Omicron. The color changes from yellow to green to represent the amino acids lipophilicity potential, ranging from 20 to −20.

In the hydrophobic top view of the original variant (Figure 9A), the hydrophobic surface is dominated by negative lipophilicity potential, and there are some positive potentials in the center of the top view. For the Alpha variant (Figure 9B), the hydrophobic surface is also dominated by negative potential, with some minor positive and neutral potentials. The negative potential dominates the Beta variant (Figure 9C), but more neutral potentials exist. The negative potential with minor neutral and positive potentials dominates the Delta variant (Figure 9D). The Gamma variant (Figure 9E) is dominated by negative potential. The Omicron variant (Figure 9F) is mainly dominated by negative potential, while some positive potentials are scattered around. Generally, the top view of the hydrophobic surface is similar among these variants.

**Figure 10:**
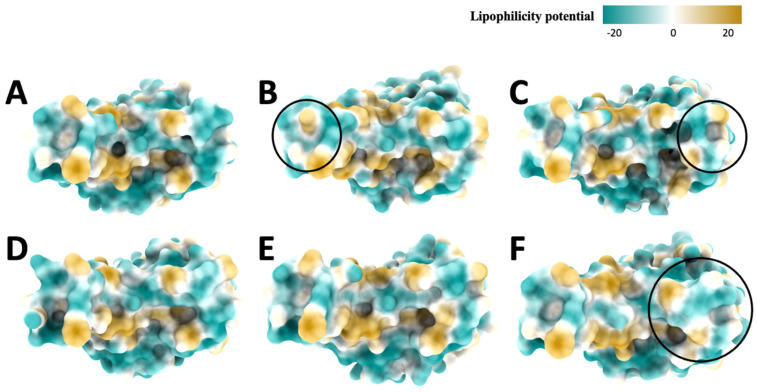
Hydrophobic bottom view, (**A**): Original variant; (**B**): Alpha variant; (**C**): Beta variant; (**D**): Delta variant; (**E**): Gamma variant; (**F**): Omicron variant. The circled area indicates the differences in shape among variants.

In the bottom view of the original variant (Figure 10A) hydrophobic surface, the area is covered close to evenly by negative and positive potentials along with neutral potentials, and the pocket has slightly positive potential and is close to neutral. The Alpha variant (Figure 10B) is evenly covered by positive and negative potentials and some neural potentials. We also noticed that the Alpha variant’s hydrophobic structure differs slightly from the others. We noticed the left part of the receptor is not inward like the other variants (and the outward part has high lipophilicity), so the Alpha variant is more likely to bind to hydrophobic targets other than the desired target. The Beta variant (Figure 10C) has more negative than positive potentials, along with some neutral potentials. Structurally, the Beta variant’s rightmost part is different from other variants. For the Delta variant (Figure 10D), the hydrophobic surface combined negative and positive potentials, mostly even with some neutral potentials. We notice that the leftmost pocket shape differs from the other variants; the shape looks like two pockets instead of one. For the Gamma variant (Figure 10E), the positive and negative potentials are almost evenly distributed, and the potentials of the pocket have a slightly positive potential. For the Omicron variant (Figure 10F), the hydrophobic surface is mainly distributed even by the negative and positive potentials along with some neutral potentials, and the pocket has slightly positive potentials and close to neutral potentials.

Among the bottom view of these variants’ hydrophobic surface, the distribution of the hydrophobic surface is primarily positive and negative potentials, and there is no apparent trend of the dominant potentials, which contributes to not making the area of binding receptor highly hydrophilic or hydrophobic and make these variants to be steady. Interestingly, the pocket area is also mostly evenly distributed by negative, positive, and neutral potentials around the pocket area.

### 2.6. Binding Affinity Calculations

**Figure 11:**
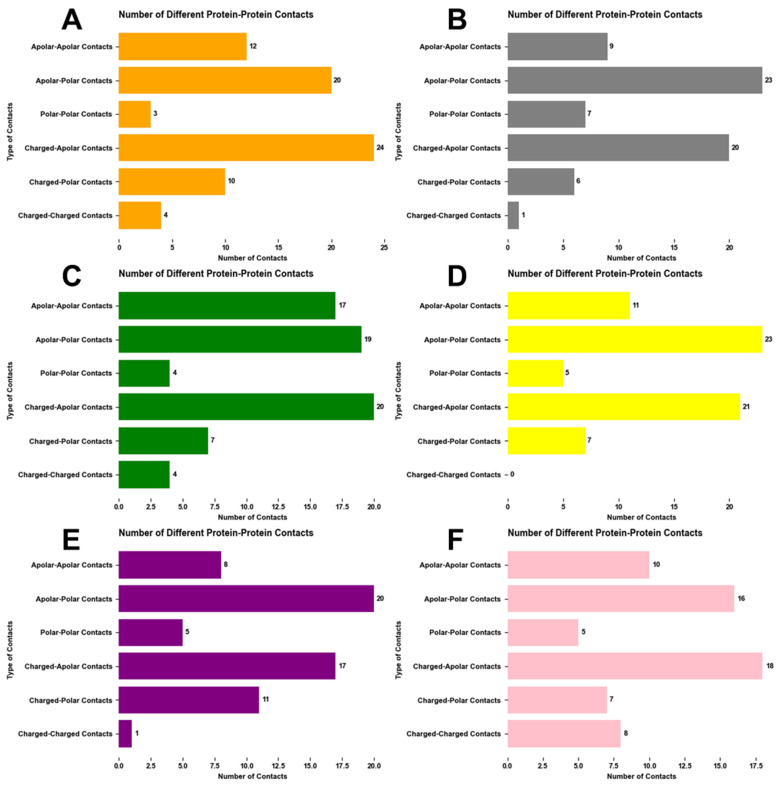
The analysis of protein-protein contacts (PPCs) across spike proteins of the SARS-CoV-2 variants. (**A**) Original (orange); (**B**) Alpha (gray); (**C**) Beta (green); (**D**) Gamma (yellow); (**E**) Delta (purple); (**F**) Omicron (pink). Each graph shows the number of different protein-protein contacts in spike proteins, including charged–charged contacts, charged–polar contacts, charged–apolar contacts, polar–polar contacts, apolar–polar contacts, and apolar–apolar contacts.

We deployed the PRODIGY tool to analyze the binding affinities and interaction patterns of SARS-CoV-2 variants with ACE2 receptors. Intermolecular contacts were grouped into six classes based on their polar/apolar/charged types, i.e., charged–charged, charged–polar, charged–apolar, polar–polar, polar–apolar, and apolar–apolar contacts, as shown in Figure 11. Moreover, PRODIGY provided information about Non-Interacting Surface (NIS) properties (Table 2). The binding affinities and dissociation constants were calculated using Equation (2), represented in Table 3. This equation effectively quantifies the impact of various interfacial contacts and surface properties on overall binding affinity. For instance, positive coefficients observed for charged–charged and polar–polar interactions indicate their favorable contributions via such mechanisms as salt bridges and hydrogen bonds. On the other hand, negatively signed coefficients for charged–apolar and polar–apolar interactions emphasize their destabilizing effects due to misfit in complementary properties. Additionally, the equation also considers non-interacting surfaces where apolar or charged characteristics can hinder or facilitate solvation or ionic interactions, respectively, thus highlighting how complex the molecular structure–dynamics relationship is.

The Original strain (Figure 11A) has 4 charged–charged, 24 charged–apolar, and 12 apolar–apolar PPCs, resulting in substantial negative Δ*G* (−12.7 kcal/mol), indicating a strong binding affinity. The 33.33% apolar and 27.02% charged NIS residues, based on the PRODIGY model, create a balanced interface for binding. The Alpha variant (Figure 11B) becomes more hydrophobic with one charged–charged PPC and 41.31% apolar NIS residues, which lowers the binding energy to −11.1 kcal/mol. Beta (Figure 11C) keeps 4 charged–charged PPCs and increases apolar–apolar PPCs to 17 with 37.1% apolar NIS residues, slightly lowering binding energy to −11.6 kcal/mol. Gamma (Figure 11D) has no charged–charged PPCs but lots of charged–apolar PPCs (21) and 35.22% apolar NIS residues, maintaining high binding energy at −12.2 kcal/mol, close to the Original. Delta (Figure 11E) shifts to more charged–polar interactions (11) and fewer total PPCs (62) and shows a slight loss in binding energy at −11 kcal/mol. Omicron (Figure 11F) has the highest charged–charged PPCs at 8 but slightly lower total PPC count (64) and 35.26% apolar NIS residues, with a solid binding energy of −11 kcal/mol.

The PRODIGY model explains binding affinity changes in viral variants. Mutations lead to different protein-protein contacts (PPCs) and non-interacting surface (NIS) residue compositions. According to the PRODIGY model’s predictions, such mutations can either synergistically improve or antagonistically reduce the bond strength.

## 3. Methods

### 3.1. Preparing Protein Structures

As the initial step of this study, the protein structures of the Original, Alpha, Beta, Gamma, Delta, and Omicron variants of the SARS-CoV-2 coronavirus were acquired from the Protein Data Bank, with 6VW1 [54], 7FEM [55], 7VXD [56], 7NXC [57], 7V8B [58], and 7U0N [59] being their PDB-IDs, respectively. The structural alignment process was crucial to ensure accurate comparisons using the Chimera tool [60]. Chimera was used to align all the variants’ structures with an original virus (6VW1), where every variant’s length was trimmed to the same size as the Original and was compared (Figure 1). This structure alignment can help gain insights into these variants’ evolutionary history. Structural similarities can help infer common ancestry and evolutionary relationships [61]. It also helped focus on RBD, responsible for interacting with the hACE2 protein. Eliminating the part of the structure not aligned with the original virus decreased the computational time.

### 3.2. Structural Comparison

The protein structures SARS-CoV-2 and ACE2 (PDB ID 6VW1) and its variants Alpha (PDB ID 7FEM), Beta (PDB ID 7VXD), Gamma (PDB ID 7NXC), Delta (PDB ID 7V8B), and Omicron (PDB ID 7U0N) were downloaded from the Protein Data Bank (PDB) [62]. Before performing the structural comparison, we resized the Alpha, Beta, and Omicron protein structures for better comparison performance. Section 2.1 compares the Original and Alpha protein structures, Alpha and Beta, Beta and Gamma, Gamma and Delta, and Delta and Omicron. We found the structural differences for each pair of the variant comparisons by comparing the DNA sequences. Then, we colorized the amino acid side chains of the DNA sequence differences in a ball stick shape. The amino acid side chains play a crucial role in proteins’ folding and final 3D structure, which is also helpful in observing protein structural differences and analyzing hydrophobic interactions.

### 3.3. Performing Molecular Dynamic Simulations

Protein Structure Files (PSFs) were generated using the Visual Molecular Dynamics 1.9.4 (VMD) tool [63] for each variant, serving as a crucial component to define the atomistic details of the protein structures, including their composition and connectivity. PSFs contain information about the atoms, bonds, angles, and dihedrals within the protein structure. To simulate the interaction between each variant with hACE2, MD simulation was performed using the NAMD2 tool. Each simulation was performed at 300 k temperature and 2000 minimization steps, followed by 10 million degrees. It resulted in 1000 frames for each simulation of 10 ns. The first 5 ns of the simulation is unstable, So the results of the last 5 ns were considered for a more accurate result [36].

### 3.4. Salt Bridge and Hydrogen Bond Analysis from MD Simulation Results

Using VMD, data on salt bridges and hydrogen bonds were extracted. Hydrogen bonds with occupancies that were more significant than 75% were considered for the analysis (Figure 2). Hydrogen bond data were generated for the last 500 frames, keeping the Donor-Acceptor distance at 4.0 Å. The number of hydrogen bonds formed, their length, and the strength of the interactions were quantified for each variant. Salt bridges were removed with a cutoff at 3.2 Å, and the data from the last 500 frames of the simulation were utilized for the analysis (Figure 3). For hydrogen bonds, the cutoff was set at 4 Å. A total of 3.2 Å was used as a base reference line to determine the strength of the salt bridges and the salt bridges that were distanced consistently above 6 Å were excluded as they do not contribute much to the stability of the interaction. 

### 3.5. Visualizing Salt Bridges and Hydrogen Bonds Data

The salt bridges and hydrogen bonds data, extracted from MD simulation results, were converted into separate values formatted (CSV) files using Python script. These CSV files were then utilized to visualize the results using Microsoft Excel (Figure 2 and Figure 3). Considering the salt bridge data (Figure 2), a line chart was used to visualize the data where each graph represents a salt bridge formed during the interaction, and a standard horizontal black line at 3.2 Å was used to determine the strength of the salt bridge. Hydrogen bonds (Figure 3) were represented using bar graphs where each bar represents the hydrogen bond formed and occupancy of the respective hydrogen bond. The colors for each salt bridge and hydrogen bond were determined to be the same as those for the variants.

### 3.6. Electrostatic Surface

To show the electrostatic surface, we calculated the protein electrostatic potential of each variant by solving the Poisson–Boltzmann equation using Delphi and visualized it in ChimeraX. The Poisson–Boltzmann equation (PBE) is as follows:

∇• [ǫ (*r*) ∇φ (*r*)] =−4πρ (*r*) +ǫ (*r*) κ (1)

where φ(*r*) is the electrostatic potential, ǫ (*r*) is the dielectric distribution, ρ (*r*) is the charge density based on the atomic structures, κ is the Debye–Huckel parameter, *kB* is the Boltzmann constant, and *T* is the temperature.

The calculated electrostatic potentials by Delphi were based on the electrostatic potential maps. The electrostatic potential maps were computed using the PDB2PQR server and APBS tools; we set the pH to 7.0 in the PDB2PQR configuration. After we had the electrostatic potential maps, we colored the protein surface by the electrostatic potential, with red representing the negative charges and blue representing the positive charges. We set the color scale range between −10 kT/Å and 10 kT/Å.

### 3.7. Hydrophobic Surface

The hydrophobicity of protein is determined by the hydrophobicity of the amino acids that compose it [64]. The kd hydrophobicity is automatically assigned to amino acid residues as an attribute with the Kyte and Doolittle hydrophobicity scale values [65]. Looking at the hydrophobicity distribution of a protein can help us better understand the folding of proteins.

### 3.8. Binding Affinity Calculation

The resulting docking data of each variant were processed and analyzed using the tools of PRODIGY software (https://rascar.science.uu.nl/prodigy/, accessed on 1 February 2023) Field [66]. Within PRODIGY, a simple but robust descriptor of binding affinity is implemented based solely on the structural properties of protein–protein complexes [67]. Using the protein–protein critical affinity benchmark of Kastritis et al., 2011 [44], PRODIGY directly correlates the number of interfacial contacts (ICs) in a protein–protein complex and the binding strength. Its predictor is, therefore, based on a simple linear regression of ICs and some of the properties of the non-interacting surfaces (NISs), which have been shown to influence the binding affinity by Kastritis et al., 2014 [68].

(2) $$\begin{array}{cc} \Delta G_{predicted}= & -0.09459\;{ICs}_{charged/charged}-0.1007\;{ICs}_{charged/apolar}+0.19577\;{ICs}_{polar/polar}\\ & -0.22671\;{ICs}_{polar/apolar}+0.18681\;{\%NIS}_{apolar}+0.3810\;{\%NIS}_{charged}-15.9433\; \end{array}$$

where *ICs_xxx/yyy_* is the number of Interfacial Contacts found at the interface between Interactor1 and Interactor2 classified according to the polar/apolar/charged nature of the interacting residues (i.e., *ICs_charged/apolar_* is the number of ICs between charged and apolar residues). Two residues are defined in contact if any of their heavy atoms is within 5.5 Å.

Based on the predicted binding affinity (Δ*G*) according to Equation (1), the dissociation constant (*K_d_*) is calculated via the following formula:

(3) $$\Delta G=RT\;\ln K_{d\;}$$

where *R* is the ideal gas constant (in kcal K^−1^mol^−1^), *T* is the temperature (in K), and Δ*G* is the predicted free energy. By default, the temperature is set at 298.15 K (25.0 °C).

## 4. Conclusions

Our structural comparison reveals significant mutations between the Delta and Omicron variants, with fewer mutations occurring between the Alpha and Beta, Beta and Gamma variants. The RMSD calculations indicate that the Original and Omicron variants are most similar. Hydrogen bond analysis shows that hydrogen bonds significantly contribute to the overall stability of protein–protein interactions between SARS-CoV-2 variants and the hACE2 protein. From our observation, the Gamma variant has the highest hydrogen bond occupancy among all the variants with the top three hydrogen bonds, Arg559-Glu564, Arg115-Glu182, and Arg559-Glu564. This observation indicates that the Gamma variant has the strongest interaction with hACE2.

Salt bridge analysis from MD simulations revealed that the Alpha and Delta variants have the highest number of solid salt bridges, while the Gamma variant shows weaker interactions with only one pair of strong salt bridges. Glutamic acid (Glu) and Lysine (Lys) frequently form stable bridges, whereas Glutamic acid paired with Arginine forms fewer sturdy bridges. This suggests that not all amino acids equally contribute to the stability of salt bridges and the protein’s structural integrity. Key residues such as Arginine and Aspartic acid are crucial in forming high-occupancy hydrogen bonds, highlighting potential targets for antiviral strategies. Therapeutic agents can be developed to disrupt these interactions, breaking the connection between SARS-CoV-2 variants and hACE2.

The electrostatic potential distribution shows high similarity between the Original and Omicron variants. Given that human ACE2 is overall negative, variants with predominantly positive potential have high binding affinity, making humans more easily affected. The pocket area, an active mutation site, shows varying potentials: Original’s pocket is more negative than Delta’s, turns negative again from Delta to Gamma, and becomes neutral with slightly negative potential from Gamma to Omicron. These variations suggest significant implications for drug design and effectiveness. Supported by salt bridge results from MD simulations, these insights are valuable for developing effective therapeutic drugs.

Lipophilicity distribution analysis shows similar hydrophilic properties for the Beta, Gamma, and Omicron variants, consistent with their electrostatic surfaces. The distribution of hydrophobic surfaces is balanced, contributing to the stability of the variants. These analyses are crucial for drug design, providing insights into the variants’ hydrophobic features.

Non-interacting surface (NIS) analysis and binding affinity calculations show that mutations lead to different protein–protein contacts (PPCs) and NIS residue compositions. Either apolar–polar contacts (APCs) or charged–apolar contacts (CACs) are dominant in each variant. Omicron has the highest charged–charged PPCs. Throughout the whole mutation process (from Original to Omicron), PPCs, charged–polar contacts tend to have similar amounts. Apolar–apolar contacts are unstable, with APCs and CACs contributing most to protein–protein interaction.

This work enhances the collective scientific understanding of the SARS-CoV-2 virus and its variants, with potential implications for therapeutics and public health measures. The study highlights the importance of using computational tools to uncover the complex interplay of molecular interactions in biological systems, paving the way for future research.

## Figures and Tables

**Table 1:** Number of strong and weak salt bridges for each variant.

Variant	Strong (Pair(s))	Weak (Pairs)
Original	1	2
Alpha	2	
Beta	1	2
Gamma	1	
Delta	2	
Omicron	1	3

**Table 2:** Percentage of non-interacting surface (NIS) residues for each variant.

Variant	% of Apolar NIS Residues	% of Charged NIS Residues
Original	33.33	27.02
Alpha	41.31	22.36
Beta	37.1	24.2
Gamma	35.22	25.09
Delta	35.99	25.64
Omicron	35.26	26.14

**Table 3:** The predicted binding affinity (Δ*G*) and the dissociation constant ( $K_{d}$ ) for each.

Variant	Δ*G* (kcal/mol)	$K_{d}$ (M) at 25.0 °C
Original	−12.7	4.70 × 10^−10^
Alpha	−11.1	7.50 × 10^−9^
Beta	−11.6	3.20 × 10^−9^
Gamma	−12.2	1.10 × 10^−9^
Delta	−11	8.10 × 10^−9^
Omicron	−11	9.30 × 10^−9^

## Data Availability

The data are not publicly available because all the Original Data we got are from Protein Data Bank, which is open to public.

## References

[1] Hu B., Guo H., Zhou P., Shi Z.-L. (2021). Characteristics of SARS-CoV-2 and COVID-19. Nat. Rev. Microbiol..

[2] Carabelli A.M., Peacock T.P., Thorne L.G., Harvey W.T., Hughes J., Peacock S.J., Barclay W.S., de Silva T.I., Towers G.J., COVID-19 Genomics UK Consortium (2023). SARS-CoV-2 variant biology: Immune escape, transmission and fitness. Nat. Rev. Microbiol..

[3] Johansson M.A., Quandelacy T.M., Kada S., Prasad P.V., Steele M., Brooks J.T., Slayton R.B., Biggerstaff M., Butler J.C. (2021). SARS-CoV-2 transmission from people without COVID-19 symptoms. JAMA Netw. Open.

[4] Matheson N.J., Lehner P.J. (2020). How does SARS-CoV-2 cause COVID-19?. Science.

[5] Mohammadi M., Shayestehpour M., Mirzaei H. (2021). The impact of spike mutated variants of SARS-CoV2 [Alpha, Beta, Gamma, Delta, and Lambda] on the efficacy of subunit recombinant vaccines. Braz. J. Infect. Dis..

[6] Rahimi A., Mirzazadeh A., Tavakolpour S. (2021). Genetics and genomics of SARS-CoV-2: A review of the literature with the special focus on genetic diversity and SARS-CoV-2 genome detection. Genomics.

[7] Guruprasad L. (2021). Human SARS CoV-2 spike protein mutations. Proteins Struct. Funct. Bioinform..

[8] Walensky R.P., Walke H.T., Fauci A.S. (2021). SARS-CoV-2 variants of concern in the United States—Challenges and opportunities. JAMA.

[9] Wise J. (2020). COVID-19: New coronavirus variant is identified in UK. BMJ.

[10] Tegally H., Wilkinson E., Giovanetti M., Iranzadeh A., Fonseca V., Giandhari J., Doolabh D., Pillay S., San E.J., Msomi N. (2021). Detection of a SARS-CoV-2 variant of concern in South Africa. Nature.

[11] Faria N.R., Mellan T.A., Whittaker C., Claro I.M., Candido D.d.S., Mishra S., Crispim M.A., Sales F.C., Hawryluk I., McCrone J.T. (2021). Genomics and epidemiology of the P. 1 SARS-CoV-2 lineage in Manaus, Brazil. Science.

[12] Abdool Karim S.S., de Oliveira T. (2021). New SARS-CoV-2 variants—Clinical, public health, and vaccine implications. N. Engl. J. Med..

[13] Sabino E.C., Buss L.F., Carvalho M.P., Prete C.A., Crispim M.A., Fraiji N.A., Pereira R.H., Parag K.V., da Silva Peixoto P., Kraemer M.U. (2021). Resurgence of COVID-19 in Manaus, Brazil, despite high seroprevalence. Lancet.

[14] Salvatore M., Bhattacharyya R., Purkayastha S., Zimmermann L., Ray D., Hazra A., Kleinsasser M., Mellan T., Whittaker C., Flaxman S. (2021). Resurgence of SARS-CoV-2 in India: Potential role of the B. 1.617. 2 (Delta) variant and delayed interventions. medRxiv.

[15] Fan Y., Li X., Zhang L., Wan S., Zhang L., Zhou F. (2022). SARS-CoV-2 Omicron variant: Recent progress and future perspectives. Signal Transduct. Target. Ther..

[16] Tao K., Tzou P.L., Nouhin J., Gupta R.K., de Oliveira T., Kosakovsky Pond S.L., Fera D., Shafer R.W. (2021). The biological and clinical significance of emerging SARS-CoV-2 variants. Nat. Rev. Genet..

[17] Nguyen H.L., Lan P.D., Thai N.Q., Nissley D.A., O’Brien E.P., Li M.S. (2020). Does SARS-CoV-2 bind to human ACE2 more strongly than does SARS-CoV?. J. Phys. Chem. B.

[18] Pandey R.K., Ojha R., Prajapati V.K. (2020). Wet-Lab Approaches to Determine Three-Dimensional Structures of Proteins. Frontiers in Protein Structure, Function, and Dynamics.

[19] Ali A., Vijayan R. (2020). Dynamics of the ACE2–SARS-CoV-2/SARS-CoV spike protein interface reveal unique mechanisms. Sci. Rep..

[20] Wu C., Liu Y., Yang Y., Zhang P., Zhong W., Wang Y., Wang Q., Xu Y., Li M., Li X. (2020). Analysis of therapeutic targets for SARS-CoV-2 and discovery of potential drugs by computational methods. Acta Pharm. Sin. B.

[21] Sobitan A., Mahase V., Rhoades R., Williams D., Liu D., Xie Y., Li L., Tang Q., Teng S. (2021). Computational saturation mutagenesis of SARS-CoV-1 spike glycoprotein: Stability, binding affinity, and comparison with SARS-CoV-2. Front. Mol. Biosci..

[22] Xie Y., Karki C.B., Chen J., Liu D., Li L. (2021). Computational study on DNA repair: The roles of electrostatic interactions between uracil-DNA glycosylase (UDG) and DNA. Front. Mol. Biosci..

[23] Xie Y., Li L. (2022). Computational Study on E-Hooks of Tubulins in the Binding Process with Kinesin. Int. J. Mol. Sci..

[24] Xie Y., Li L. (2022). Computational Study on the Electrostatic Interactions between Uracil-DNA Glycosylase (UDG) and DNA. FASEB J..

[25] Xie Y. (2022). Developing and Applying Computational Algorithms to Reveal Health-Related Biomolecular Interactions. Ph.D. Thesis.

[26] Guo W., Xie Y., Lopez-Hernandez A.E., Sun S., Li L. (2021). Electrostatic features for nucleocapsid proteins of SARS-CoV and SARS-CoV-2. Math. Biosci. Eng..

[27] Lopez-Hernandez A.E., Xie Y., Guo W., Li L. (2021). The electrostatic features of dengue virus capsid assembly. J. Comput. Biophys. Chem..

[28] Sun S., Lopez J.A., Xie Y., Guo W., Liu D., Li L. (2022). HIT web server: A hybrid method to improve electrostatic calculations for biomolecules. Comput. Struct. Biotechnol. J..

[29] Sun S., Xu H., Xie Y., Sanchez J.E., Guo W., Liu D., Li L. (2023). HIT-2: Implementing machine learning algorithms to treat bound ions in biomolecules. Comput. Struct. Biotechnol. J..

[30] Sun S., Karki C., Xie Y., Xian Y., Guo W., Gao B.Z., Li L. (2021). Hybrid method for representing ions in implicit solvation calculations. Comput. Struct. Biotechnol. J..

[31] Rodriguez G., Martinez G.S., Negrete O.D., Sun S., Guo W., Xie Y., Li L., Xiao C., Ross J.A., Kirken R.A. (2023). JAK3 Y841 Autophosphorylation Is Critical for STAT5B Activation, Kinase Domain Stability and Dimer Formation. Int. J. Mol. Sci..

[32] Xie Y., Li L. Multi-Scale Computational Study on SARS-CoV and SARS-CoV-2. Proceedings of the APS March Meeting Abstracts.

[33] Xie Y., Guo W., Lopez-Hernadez A., Teng S., Li L. (2022). The pH effects on SARS-CoV and SARS-CoV-2 spike proteins in the process of binding to hACE2. Pathogens.

[34] Sun S., Rodriguez G., Xie Y., Guo W., Hernandez A.E.L., Sanchez J.E., Kirken R.A., Li L. (2023). Phosphorylation of Tyrosine 841 Plays a Significant Role in JAK3 Activation. Life.

[35] Xie Y., Du D., Karki C.B., Guo W., Lopez-Hernandez A.E., Sun S., Juarez B.Y., Li H., Wang J., Li L. (2020). Revealing the mechanism of SARS-CoV-2 spike protein binding with ACE2. Comput. Sci. Eng..

[36] Xie Y., Karki C.B., Du D., Li H., Wang J., Sobitan A., Teng S., Tang Q., Li L. (2020). Spike proteins of SARS-CoV and SARS-CoV-2 utilize different mechanisms to bind with human ACE2. Front. Mol. Biosci..

[37] Xian Y., Xie Y., Silva S.M., Karki C.B., Qiu W., Li L. (2021). StructureMan: A structure manipulation tool to study large scale biomolecular interactions. Front. Mol. Biosci..

[38] Guo W., Sun S., Sanchez J.E., Lopez-Hernandez A.E., Ale T.A., Chen J., Afrin T., Qiu W., Xie Y., Li L. (2022). Using a comprehensive approach to investigate the interaction between Kinesin-5/Eg5 and the microtubule. Comput. Struct. Biotechnol. J..

[39] Salas G.G.S., Hernandez A.E.L., He J., Karki C., Xie Y., Sun S., Xian Y., Li L. (2019). Using computational approaches to study dengue virus capsid assembly. Comput. Math. Biophys..

[40] Xie Y. (2021). Applying Computational Methods to Study the Interactions Between Sars-Cov-2 and hACE2. Master’s Thesis.

[41] Mahase V., Sobitan A., Johnson C., Cooper F., Xie Y., Li L., Teng S. (2020). Computational analysis of hereditary spastic paraplegia mutations in the kinesin motor domains of KIF1A and KIF5A. J. Theor. Comput. Chem..

[42] Karki C., Xian Y., Xie Y., Sun S., Lopez-Hernandez A.E., Juarez B., Wang J., Sun J., Li L. (2020). A computational model of ESAT-6 complex in membrane. J. Theor. Comput. Chem..

[43] Cui Y., Cao Z., Xie Y., Jiang X., Tao F., Chen Y.V., Li L., Liu D. Dg-labeler and dgl-mots dataset: Boost the autonomous driving perception. Proceedings of the IEEE/CVF Winter Conference on Applications of Computer Vision.

[44] Anik F.I., Sakib N., Shahriar H., Xie Y., Nahiyan H.A., Ahamed S.I. (2023). Unraveling a blockchain-based framework towards patient empowerment: A scoping review envisioning future smart health technologies. Smart Health.

[45] Khan A., Gui J., Ahmad W., Haq I., Shahid M., Khan A.A., Shah A., Khan A., Ali L., Anwar Z. (2021). The SARS-CoV-2 B. 1.618 variant slightly alters the spike RBD–ACE2 binding affinity and is an antibody escaping variant: A computational structural perspective. RSC Adv..

[46] Kumar S., Thambiraja T.S., Karuppanan K., Subramaniam G. (2022). Omicron and Delta variant of SARS-CoV-2: A comparative computational study of spike protein. J. Med. Virol..

[47] Shi J., Wen Z., Zhong G., Yang H., Wang C., Huang B., Liu R., He X., Shuai L., Sun Z. (2020). Susceptibility of ferrets, cats, dogs, and other domesticated animals to SARS–coronavirus 2. Science.

[48] Jumper J., Evans R., Pritzel A., Green T., Figurnov M., Ronneberger O., Tunyasuvunakool K., Bates R., Žídek A., Potapenko A. (2021). Highly accurate protein structure prediction with AlphaFold. Nature.

[49] Mirza M.U., Froeyen M. (2020). Structural elucidation of SARS-CoV-2 vital proteins: Computational methods reveal potential drug candidates against main protease, Nsp12 polymerase and Nsp13 helicase. J. Pharm. Anal..

[50] Barroso da Silva F.L., Giron C.C., Laaksonen A. (2022). Electrostatic features for the receptor binding domain of SARS-COV-2 wildtype and its variants. Compass to the severity of the future variants with the charge-rule. J. Phys. Chem. B.

[51] Hristova S.H., Zhivkov A.M. (2024). Three-Dimensional Structural Stability and Local Electrostatic Potential at Point Mutations in Spike Protein of SARS-CoV-2 Coronavirus. Int. J. Mol. Sci..

[52] Aksenova A.Y., Likhachev I.V., Grishin S.Y., Galzitskaya O.V. (2022). The increased amyloidogenicity of spike RBD and pH-dependent binding to ACE2 may contribute to the transmissibility and pathogenic properties of SARS-CoV-2 omicron as suggested by in silico study. Int. J. Mol. Sci..

[53] Hristova S.H., Zhivkov A.M. (2023). Omicron Coronavirus: pH-Dependent Electrostatic Potential and Energy of Association of Spike Protein to ACE2 Receptor. Viruses.

[54] Babaeekhou L., Ghane M., Abbas-Mohammadi M. (2021). In silico targeting SARS-CoV-2 spike protein and main protease by biochemical compounds. Biologia.

[55] Barre A., Klonjkowski B., Benoist H., Rougé P. (2022). How Do Point Mutations Enhancing the Basic Character of the RBDs of SARS-CoV-2 Variants Affect Their Transmissibility and Infectivity Capacities?. Viruses.

[56] Wang Y., Xu C., Wang Y., Hong Q., Zhang C., Li Z., Xu S., Zuo Q., Liu C., Huang Z. (2021). Conformational dynamics of the Beta and Kappa SARS-CoV-2 spike proteins and their complexes with ACE2 receptor revealed by cryo-EM. Nat. Commun..

[57] Ovchynnykova O., Kapusta K., Sizochenko N., Sukhyy K.M., Kolodziejczyk W., Hill G.A., Saloni J. (2022). Homology Modeling and Molecular Dynamics-Driven Search for Natural Inhibitors That Universally Target Receptor-Binding Domain of Spike Glycoprotein in SARS-CoV-2 Variants. Molecules.

[58] Liu H., Wei P., Kappler J.W., Marrack P., Zhang G. (2022). SARS-CoV-2 variants of concern and variants of interest receptor binding domain mutations and virus infectivity. Front. Immunol..

[59] Wang Q., Guo Y., Iketani S., Nair M.S., Li Z., Mohri H., Wang M., Yu J., Bowen A.D., Chang J.Y. (2022). Antibody evasion by SARS-CoV-2 Omicron subvariants BA. 2.12. 1, BA. 4 and BA. 5. Nature.

[60] Pettersen E.F., Goddard T.D., Huang C.C., Couch G.S., Greenblatt D.M., Meng E.C., Ferrin T.E. (2004). UCSF Chimera—A visualization system for exploratory research and analysis. J. Comput. Chem..

[61] Song N., Joseph J.M., Davis G.B., Durand D. (2008). Sequence similarity network reveals common ancestry of multidomain proteins. PLoS Comput. Biol..

[62] Dolinsky T.J., Nielsen J.E., McCammon J.A., Baker N.A. (2004). PDB2PQR: An automated pipeline for the setup of Poisson–Boltzmann electrostatics calculations. Nucleic Acids Res..

[63] Humphrey W., Dalke A., Schulten K. (1996). VMD: Visual molecular dynamics. J. Mol. Graph..

[64] Mahn A., Lienqueo M.E., Salgado J.C. (2009). Methods of calculating protein hydrophobicity and their application in developing correlations to predict hydrophobic interaction chromatography retention. J. Chromatogr. A.

[65] Moelbert S., Emberly E., Tang C. (2004). Correlation between sequence hydrophobicity and surface-exposure pattern of database proteins. Protein Sci..

[66] Xue L.C., Rodrigues J.P., Kastritis P.L., Bonvin A.M., Vangone A. (2016). PRODIGY: A web server for predicting the binding affinity of protein–protein complexes. Bioinformatics.

[67] Vangone A., Bonvin A.M. (2015). Contacts-based prediction of binding affinity in protein–protein complexes. eLife.

[68] Kastritis P.L., Rodrigues J.P., Folkers G.E., Boelens R., Bonvin A.M. (2014). Proteins feel more than they see: Fine-tuning of binding affinity by properties of the non-interacting surface. J. Mol. Biol..

